# Updating the fungal infection-mammalian selection hypothesis at the end of the Cretaceous Period

**DOI:** 10.1371/journal.ppat.1008451

**Published:** 2020-07-16

**Authors:** Arturo Casadevall, Chris Damman

**Affiliations:** 1 Department of Molecular Microbiology and Immunology, Johns Hopkins School of Public Health, Baltimore, Maryland, United States of America; 2 The Bill & Melinda Gates Foundation, Seattle, Washington, United States of America; 3 University of Washington, Seattle, Washington, United States of America; Geisel School of Medicine at Dartmouth, UNITED STATES

In 2005, one of us proposed that a fungal bloom at the end of the Cretaceous Period would have favored the selection of the endothermic mammals over ectothermic reptiles, which eventually led to the great mammalian radiation and the replacement of the Cretaceous reptilian megafauna with the mammalian megafauna of the Tertiary or Paleogene Period [1]. This idea, which we now name the “fungal infection-mammalian selection” (FIMS) hypothesis, suggested a new explanation for how the mammals came to replace reptiles as the dominant large animals after the Cretaceous Period [2], which ended 66 million years ago with a planetary cataclysm known as the Cretaceous-Paleogene extinction event. At the time, this extinction event was attributed to volcanism, a bolide impact, or some combination of both. In the ensuing decade and a half since FIMS was first proposed, considerable progress was made in understanding the events following the cataclysm at the end of the Cretaceous Period, which provide the opportunity to add refinements to this hypothesis. For example, in 2005, there was uncertainty on the temporal relationship between the bolide impact and the mass extinction event, with some estimates placing it 300,000 years earlier [3]. Today there is a growing consensus for a temporal and causative relationship between a bolide impact at Chicxulub, Mexico, in the Yucatan peninsula and the mass extinction event at the end of the Cretaceous Period [4]. This together with a greater appreciation of the planetary effects following this cataclysm [5] allow refinements and updates to the FIMS hypothesis.

## The FIMS hypothesis

The geologic record is divided into periods of which the Cretaceous is that time between the Jurassic and Paleogene that spanned a time from 145 to 66 million years ago. The Cretaceous Period came to an abrupt end with the Cretaceous-Paleogene extinction event, which saw the demise of nonavian dinosaurs and many ancient species. How did mammals become the dominant large animals in the Paleogene and later periods? A widely accepted view is that the cataclysm that marked the end of the Cretaceous Period killed off the dinosaurs creating an ecologic opening for the mammals. However, that view falters in explanative power when one considers that many species of reptiles also survived the calamity, and, given that this group of animals possesses certain advantages over mammals, it does not explain why the survivors failed to usher a second reptilian age. In this regard, the mammalian lifestyle is significantly more expensive than that of ectothermic reptiles, with field metabolic rates that are 12 to 20 times higher [6], requiring consumption of much larger amounts of food for homeostasis. The FIMS hypothesis posits that a fungal bloom following the cataclysm at the end of the Cretaceous Period selected for endothermic animals over ectothermic reptiles [1, 2]. The FIMS hypothesis was assembled from the following facts: (1) the Cretaceous-Paleogene extinction event was accompanied by planetary deforestation [7] and subsequent fungal proliferation as ensuing conditions promoted a global compost [8]; (2) the fungal bloom would have generated an abundance of fungal spores that when aerosolized would have presented large pulmonary innocula for surviving animals; (3) immunologically intact mammals are remarkably resistant to fungal diseases, which has been attributed to the combination of advanced immunity in the form of innate and adoptive immune arms and higher basal temperatures that inhibit the majority of fungal species [9]. Mammals also have receptors such as fibrinogen C containing domain 1 that have recently been shown to help control fungi in epithelial surfaces [10]. The FIMS hypothesis posits that the remarkable resistance of mammals to fungal diseases today is a consequence of fungal selection for this lifestyle at the end of the Cretaceous [2].

## The postcalamity environment

Today there is widespread agreement that a bolide impact caused the Cretaceous-Paleogene extinction event and the demise of the dinosaurian megafauna [4, 5]. The bolide struck earth in the waters off the Yucatan peninsula, setting off a planetary calamity that included blast effects, giant tsunamis, fires, and blocking of sunlight from atmospheric dust and soot that resulted in global cooling. Recent modeling of the atmospheric and climate effects following the impact suggest that continents cooled by as much as 11°C with photosynthesis being shut down from 1 to 2 years [5]. The dinosaurian fauna was presumably killed by a combination of blast effects, tsunamis, massive disruption of the food chain, which affects primarily top feeders, and rapid climate change. These cataclysmic effects could have come in a setting where many species may have already been struggling from a relatively recent eruption of the Deccan traps causing basaltic floods before the bolide impact, which could have affected the climate and released poisonous elements such as mercury [11]. Small animals capable of subterranean existence and obtaining nutrition from the ensuing detritus would have been more likely to survive to become the founders of the animal species found in subsequent epochs.

## Global cooling favors mammals over reptiles

The postcalamity environment would have presented major challenges to surviving animals, but those capable of regulating their temperatures could have had significant advantages. The combination of massive amounts of decaying vegetation, darkness, and cooler temperatures are conditions known to favor fungal proliferation, for which there is fossil evidence [8]. Fungal proliferation in the form of mushrooms growing in the decayed vegetation could have provided nutrition for surviving mammals and reptiles, which are known to eat these fungi [12, 13]. We know that mushrooms existed at the time of the bolide calamity because there are mushroom fossils dating to the early Cretaceous [14]. Insects would also have been available as a food source. Although food could have been available to temporally survive the shutdown in photosynthesis, the sudden cooling of the planet could have created major challenges for nutrition acquisition for ectothermic animals, such as reptiles, which rely on higher ambient temperatures for locomotion to find food, feeding, and digestion [15]. In contrast, mammals would have been able to effectively forage for food during cooler periods given that their higher internal temperatures permitted locomotion, food acquisition, and efficient digestion. A mushroom-rich diet may have also enhanced mammalian immunity because fungal cell walls are rich in beta-glucans, which can stimulate immune function [16]. Reptilian sex ratios are affected by ambient temperatures as evident by the fact that even a 2°C drop can skew turtle sex ratios [17]. Global cooling could have played havoc with the sex ratios among surviving reptiles, further reducing their reproductive potential. Hence, the ectothermic reptilian physiology would have constituted a severe disadvantage in a rapidly cooling climate, which would have precluded adaptation. In fact, mammalian endothermy was proposed to evolve as an adaptation for early mammals to invade the cooler nocturnal niche in search for food [18], and, if this were the case, they would have been favored in the long cold night that followed the bolide impact.

## The specter of fungal diseases

Malnutrition in surviving animals would have been complicated by the specter of infectious diseases. The postcalamity fungal bloom would have included not only mushrooms but also microscopic fungi capable of causing animal diseases. Fungal diseases are common in ectothermic animals such as frogs, salamanders, and snakes, evidenced by concurrent outbreaks affecting these groups [19], but are relatively rare in mammals as a result of endothermy and advanced immunity [2]. Fungal proliferation in decaying plant matter would have created the potential for dense spore aerosols that could have presented overwhelming infectious inoculum for ectothermic animals survivors. We know that current human fungal pathogens such as *Cryptococcus neoformans* have pathogenic strategies that emerged in deep time and date to the Cretaceous [20], implying the existence of fungal species capable of causing animal disease at the time of the calamity. To compound the troubles facing reptiles and other ectotherms, these species fight infection with induced fevers [21], in which animals raise their temperatures by insolation, but there was little or no sun in the postcalamity world as a result of light blocking by atmospheric dust, soot, and smoke [5]. Finally, reptilian eggs are susceptible to penetration and infection by fungi, which would have further decimated surviving species. In this regard, *Fusarium* spp. are known to kill developing turtle eggs [22], and fossilized hyphae have been reported in fossilized dinosaur eggs [23]. Hibernation could have provided a strategy for capable animals to weather the immediate postcalamity world and wait for the re-establishment of photosynthesis and repair of biosphere cycles. However, as illustrated by the recent discovery of white nose syndrome in bats, a fungal disease that affects bats in hibernation [24], hibernating animals with cooler temperatures could have been susceptible to fungal diseases. Today, the proposal that an increase in fungal diseases among ectothermic hosts at the end of the Cretaceous Period helped usher the age of mammals echoes with the current ongoing declines in such species from chytrid mycoses in amphibians and salamanders [25, 26], Ophidiomycosis in North American snakes [22, 27], and fusariosis in turtles [22].

## Updating FIMS

**Fig 1:**
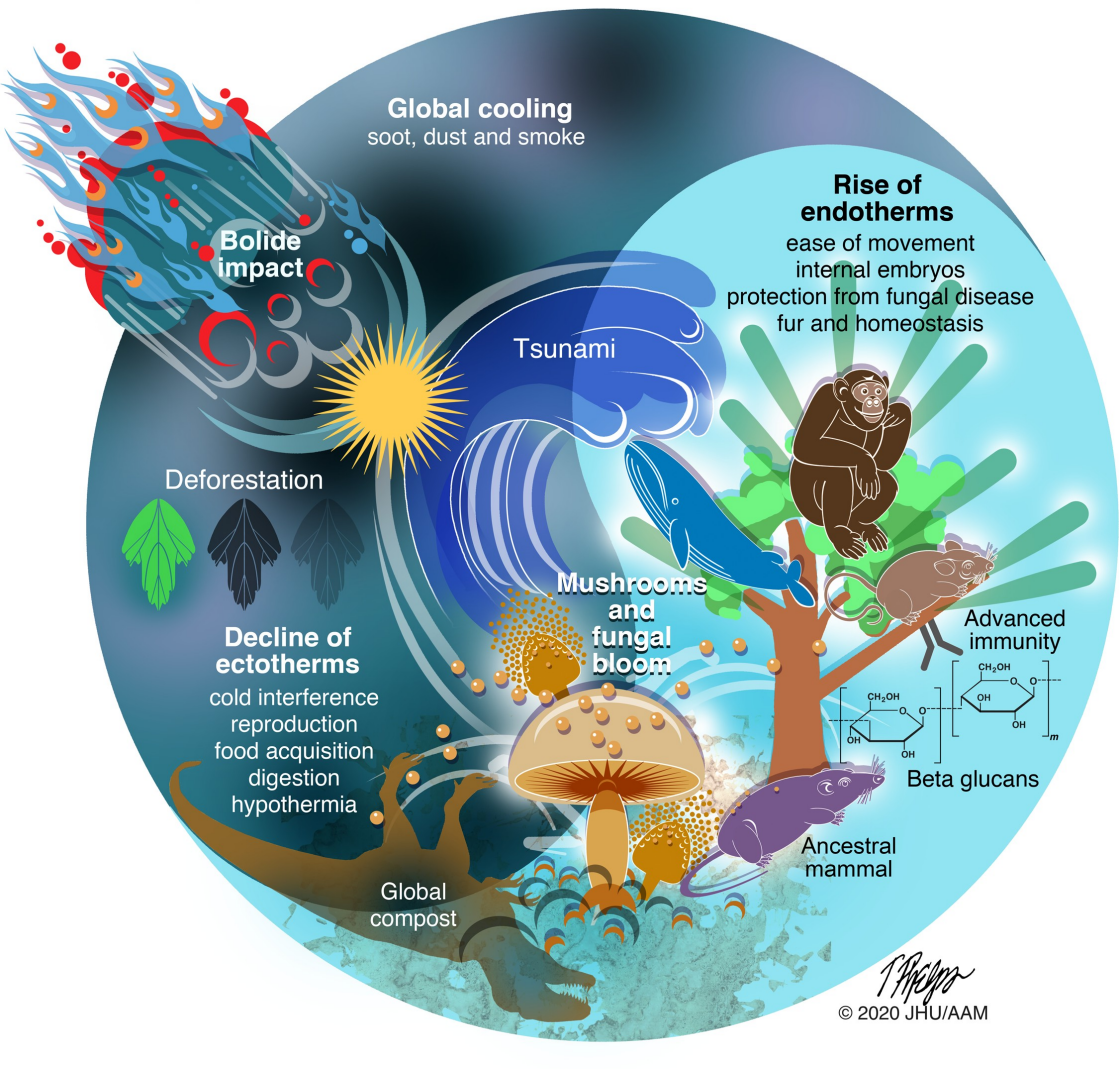
Schematic representation of the proposed events leading to the fungal selection of mammalian endotherms after the Chicxulub impact.

Since FIMS was first proposed [1], a large amount of data has accumulated that bears on the hypothesis and arguably buttresses the idea that a fungal bloom at the end of the Cretaceous Period helped usher the age of mammals (Fig 1). In the updated FIMS synthesis, mammals survived in disproportionally larger numbers than reptiles in the postimpact world because their warm body temperatures would have protected them from fungal diseases and permitted movement in the damaged biosphere to acquire and digest the available foodstuffs, including mushrooms. In contrast, the rapid global cooling would have presented great challenges to ectothermic animals such as reptiles because the cold interfered with reproduction, food acquisition, and digestion and the ensuing darkness would have precluded using insolation for inducing fever. Mammalian embryos were protected from fungal disease by the endothermy of their mothers, whereas reptilian eggs would be vulnerable to ravages from shell penetrating fungi, except possibly for buried eggs. The combination of fungal diseases and starvation would have decimated ectothermic populations while small mammals survived and prospered, such that much greater numbers of mammals survived in the postcalamity world and then populated the biosphere when the planet recovered. These more numerous mammalian survivors founded the species that then became the large mammals that populated the Paleocene, which in turn moved up in the food chain thus preventing a second age of reptiles.
